# Localizations of the septin Spn4 tagged with GFP and mEGFP in fission yeast

**DOI:** 10.17912/micropub.biology.001930

**Published:** 2025-11-26

**Authors:** Jack R. Gregory, Nicholas J. Ricottilli, Jian-Qiu Wu

**Affiliations:** 1 Department of Molecular Genetics, The Ohio State University, Columbus, Ohio, United States; 2 Molecular, Cellular, and Developmental Biology Graduate Program, The Ohio State University, Columbus, Ohio, United States; 3 Cellular, Molecular, and Biochemical Sciences Program, The Ohio State University, Columbus, Ohio, United States

## Abstract

Septins are cytoskeletal proteins crucial for cell division and many other processes. In fission yeast, septins localize to the division site during septum formation. Recently, we conducted a study that elucidates the localizations and functionalities of epitope-tagged septins. However, some questions remain outstanding. Here we assessed the impacts of monomeric mEGFP and dimeric GFP(S65T) on the septin Spn4 tagged at either of its terminus. We found that septin levels were important for its function, Spn4-mEGFP localized normally to the division site, but GFP(S65T)-Spn4 formed elongated structures ectopically, further highlighting that dimeric tags are more disruptive to septin localizations.

**Figure 1. The localization of the septin Spn4 and morphology of the strains expressing Spn4 tagged with mEGFP or GFP(S65T) at either N or C-terminus:**
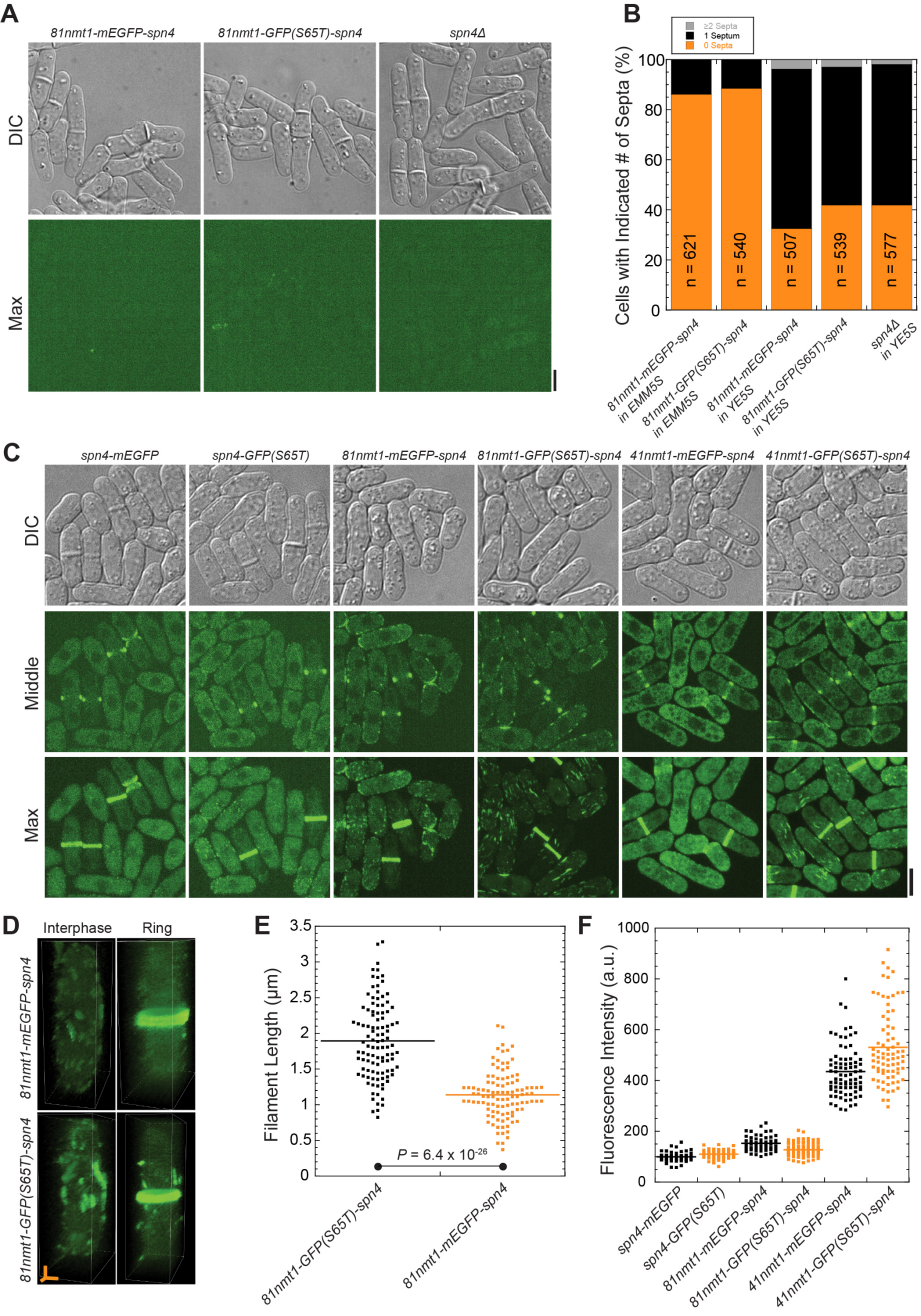
(A and B) Spn4 depletion leads to septation defects. Cells were grown in YE5S and diluted as needed to ensure exponential growth for ~36 h before imaging on a YE5S + gelatin pad. (A) Cell morphology from Differential Image Contrast (DIC) and the signal of Spn4 showing the maximal intensity projection of 25 slices with 0.3 µm spacing. (B) Quantification of the number of septa per cell from DIC images of the indicated strains. (C-F) Localization and quantification of Spn4 (filament lengths and protein levels) in the indicated strains. The indicated strains were grown in YE5S for ~12 h. Then, they were washed 3x with EMM5S and grown exponentially in EMM5S for ~24 h before imaging on an EMM5S + gelatin pad. (C) Cell morphology and the localization of Spn4 are shown via DIC, fluorescence images at the middle focal plane and the maximal intensity projection of 25 slices with 0.3 µm spacing. (D) 3D volumetric projections of *81nmt-mEGFP-spn4* and *81nmt1-GFP(S65T)-spn4* are shown for cells in interphase and during cytokinesis (Ring). (E) The three longest Spn4 filaments (excluding the septin rings) from each cell were measured. n = 105 filaments for each strain. (F) The mean Spn4 fluorescence intensity in the indicated strains after subtraction of the mean background intensity in *spn4Δ* cells. n ≥ 50 cells for each strain. Scale bars, 5&nbsp;µm. Axial bars, 2 µm.

## Description

The septin family of proteins was named after their critical functions for septum formation during cytokinesis in dividing yeast cells (Ford & Pringle, 1991; Haarer & Pringle, 1987; Hartwell, 1971; Kim et al., 1991). In budding yeast, septins first form as a nascent ring at the bud emergence and then form an hourglass with both longitudinal and latitudinal filaments; these filaments transition into a double ring during cytokinesis, before dissociating from the division site after the daughter cells separate (Byers & Goetsch, 1976; Chant et al., 1995; Marquardt et al., 2019, 2021; Rodal et al., 2005; Varela Salgado & Piatti, 2024). Septins act as an essential scaffold for the myosin-II ring and other proteins at the bud neck for cytokinesis and other processes (Bi et al., 1998; Gladfelter et al., 2001; Hartwell, 1971; Takizawa et al., 2000). In other model organisms, the septins also serve as scaffolds and/or diffusion barriers and play crucial roles in many cellular processes such as cytokinesis, plasma membrane repair, gamete formation, exocytosis, neuronal development, and others (Fares et al., 1995; Gladfelter, 2006; Kinoshita et al., 1997; Kwitny et al., 2010; Onishi et al., 2010; Prislusky et al., 2024; Singh et al., 2025; Tasto et al., 2003).

The septins accomplish these numerous functions by forming palindromic heterooligomers (An et al., 2004; Bertin et al., 2008; Cavini et al., 2021). In most species, the septins form hexamers or octamers, which are the basic building block for higher-order structures, composed of two units of each septin protein. The septins interact via their two faces— the G and NC faces— forming G to G or NC to NC interactions (Sirajuddin et al., 2007). When forming the oligomers, the C terminal tails of septins stick out from the complex (Cavini et al., 2021; Sirajuddin et al., 2007). Once formed, the oligomers then polymerize into filaments that can assemble into rings, hourglass, gauzes, meshes, bars, fibers, patches, and other structures in the cell (Bridges et al., 2014; Byers & Goetsch, 1976; Garcia et al., 2011; Hernández-Rodríguez et al., 2012; Rodal et al., 2005; Vrabioiu & Mitchison, 2006).

The fission yeast *Schizosaccharomyces pombe* is an attractive genetically tractable model organism to study septins and other fundamental cellular processes (Fantes & Hoffman, 2016; Hoffman et al., 2015; Pollard & Wu, 2010). In *S. pombe* , four septins are involved in cytokinesis, septum formation, and daughter-cell separation: Spn1 , Spn2 , Spn3 , and Spn4 (An et al., 2004; Berlin et al., 2003; Longtine et al., 1996; Tasto et al., 2003). The organization of the septins at the division site is regulated by the anillin-like protein Mid2 , while the actomyosin contractile ring is regulated by the other anillin-like protein, Mid1 (Arbizzani et al., 2022; Bähler, et al., 1998; Berlin et al., 2003; Hachet & Simanis, 2008; Lee & Wu, 2012; Petit et al., 2005; Saha & Pollard, 2012; Sohrmann et al., 1996; Tasto et al., 2003). The four septins localize to the division site as unconstricted double rings on both sides of the contractile ring during cytokinesis and septum formation (An et al., 2004; Berlin et al., 2003; Longtine et al., 1996; Tasto et al., 2003; Wu et al., 2003). They are crucial for the localizations of Rho4 GTPase, Rho guanine nucleotide exchange factor Gef3 , and the exocyst to the division site (Muñoz et al., 2014; Singh et al., 2025; Wang et al., 2015).&nbsp;

We recently reported that the localization and function of septins are susceptible to epitope tagging (Gregory et al., 2025). We found that the septins Spn1 and Spn4 expressed under their native promoters have dramatically distinct localizations on the plasma membrane with different fluorescence tags. The septins Spn1 and Spn4 tagged with tdTomato and 3HA are less functional or not functional at all, which results in localization artifacts on the plasma membrane or a loss of septin localization and function (Gregory et al., 2025). By contrast, the septins tagged with mEGFP and mYFP are more functional and localize normally to the division site (Gregory et al., 2025). Our results emphasize the need to rigorously test the functionality of epitope-tagged septins and other proteins. However, several questions were left unanswered. First, no mEGFP tagged Spn4 strains were available for comparison with GFP(S65T) tagged strains (Gregory et al., 2025), which left us uncertain if the Spn4 localization we observed was caused by weak dimeric GFP(S65T) or not (Oltrogge et al., 2014). Second, how the expression level of a single septin affects the overall septin localization and function remains unclear in fission yeast.

We first tagged Spn4 with mEGFP at its C-terminus under its native promoter and at its N-terminus with the *41nmt1* (medium strength) and *81nmt1* (weak) promoters using PCR-based gene targeting (Bähler, et al., 1998). The *nmt1* promoters are induced in medium without thiamine and repressed with thiamine (Basi et al., 1993; Maundrell, 1990). A previous study has reported that cells depleted of both Spn1 and Spn4 in the *81nmt1- spn1 81nmt1- spn4* strain resemble *spn1Δ* or *spn4Δ* cells (Wu et al., 2010). We found that *81nmt1-mEGFP- spn4* and *81nmt1-GFP(S65T)- spn4* cells grown in YE5S liquid media (with unknown amount of thiamine from yeast extract) resembled *spn4Δ* in cell morphology and septation index ( Figure 1A- B). Consistently, Spn4 signal was barely visible in these cells ( Figure 1A ). Thus, low amounts of Spn4 are not sufficient for normal septin localization and function.

Next, we compared the Spn4 localization (tagged with GFP[S65T] vs. mEGFP) and cell morphology in *81nmt1* (inducing condition), *41nmt1* (inducing condition), and C-terminally tagged *spn4* strains using confocal microscopy ( Figure 1C- F). Overall, the mEGFP-tagged strains that expressed Spn4 at different levels showed no or much less Spn4 structures on the plasma membrane or in the cytoplasm outside the septin double rings and were less likely to form elongated structures than GFP(S65T)-tagged strains ( Figure 1C- F). Interestingly, N-terminally tagged Spn4 seemed more likely to form additional structures besides the rings, whereas Spn4-mEGFP appeared only in the rings at the division site as previously reported for the more functional septin-fusion proteins (An et al., 2004; Gregory et al., 2025). Moreover, all the strains resembled the wild type in cell morphology and septation index ( Figure 1B- C).

To compare the differences between N-terminally mEGFP and GFP(S65T) tagged Spn4 strains in more detail, we observed the 3D volumetric projections of the *81nmt1* strains grown under inducing conditions ( Figure 1D ), which expressed at levels close (slightly higher) to the C-terminally tagged Spn4 ( Figure 1F ). The mEGFP-tagged strain had fewer elongated structures. In contrast, the GFP(S65T)-tagged strain had many long filaments, which even persisted after the septin rings had formed at the division site ( Figure 1D ). Indeed, quantification of the three longest Spn4 structures (not counting the septin rings) in each cell revealed that *81nmt1-GFP(S65T)- spn4* cells had significantly longer Spn4 structures ( Figure 1E ). In time-lapse microscopy, the elongated structures more likely moved or flowed longer distances at a higher velocity in *81nmt1-GFP(S65T)- spn4* than in *81nmt1-mEGFP- spn4* cells, which suggest that they are likely not associated with the plasma membrane (Extended Data). These structures were reminiscent of the septin fibers or bars that we and other labs observed previously (Davidson et al., 2016; DeMay et al., 2010; Gregory et al., 2025). It is unlikely these different Spn4 structures are due to varied Spn4 protein levels because the quantification of the mean fluorescence intensity revealed that Spn4 levels under the *81nmt1* promoter (inducing condition) were within a factor of two compared to Spn4 under its native promoter ( Figure 1F ). Moreover, in *41nmt1-GFP(S65T)- spn4* but not in *41nmt1-mEGFP- spn4* cells, Spn4 formed similar elongated structures as in *81nmt1-GFP(S65T)- spn4* cells, despite the Spn4 levels being ~3-5 times higher ( Figure 1F ). Collectively, these structures, likely artefacts caused by the non-monomeric fluorophore, reveal the range of possible structures that Spn4 , and by implication the septin proteins, can form when perturbed. It is important to note the impact of epitope tagging on Spn4 in particular because alongside Spn1 , Spn4 forms the core of the septin octamer in fission yeast (An et al., 2004). Spn4 and Spn1 are essential for septin polymerization, and perturbations to these septins may disrupt the formation of the septin complex (An et al., 2004; Gregory et al., 2025).

In conclusion, our study reveals that minimal levels of the septin Spn4 are important for septin localization and function. We find that non-monomeric GFP(S65T) tagged Spn4 is more likely to form ectopic structures outside the septin rings. We also show that both ends of Spn4 are susceptible to perturbations by an epitope tag, the N-terminus being more easily perturbed. Caution and careful assessments are required when seeking to understand any potentially novel structures formed by the septins.

## Methods

Strains constructed and used in this study are listed in Table 1. We carried out gene targeting to tag Spn4 using the PCR-based homologous recombination method as previously described (Bähler, et al., 1998; Rutherford et al., 2024). The C-terminally tagged strains were controlled by the *spn4* native promoter with the *ADH1* terminator. For N-terminal tagged strains, the *nmt1* promoters and encoding sequences for mEGFP or GFP(S65T) were inserted before the start codon of *spn4* and the *spn4* terminator was used. Growth (at 25°C), preparation, and imaging of fission yeast strains were done as previously described (Davidson et al., 2015; Gregory et al., 2025; Singh et al., 2025; Ye et al., 2025; Zhang et al., 2025). Briefly, each strain was woken up from the -80°C stocks onto YE5S plates. For strains grown in EMM5S minimal medium, strains were first grown in YE5S rich liquid medium for ~12 h, then washed 3x with EMM5S by spinning down the cells at 3,000 rpm for 30 sec and resuspending in EMM5S. The cells were then grown exponentially for ~24 h before being imaged on EMM5S + 20% gelatin pads (Davidson et al., 2016). For strains grown and imaged in YE5S, strains were grown exponentially in YE5S for ~36 h, &nbsp;and diluted twice a day to keep the cells in log phase of growth. Then, they were imaged on YE5S + 20% gelatin pads, after being collected by centrifugation at 3,000 rpm for 30 sec. To protect the cells from free radicals during imaging, n-propyl gallate (PG) was used at a final concentration of 5 µM. We imaged the cells at ~23°C on a Nikon CSU-W1 SoRa spinning disk confocal microscope with Hamamatsu ORCA Quest qCMOS camera C15550 on Nikon Eclipse Ti2 microscope with Plan Apo λD 100x/1.45 numerical aperture (NA) oil objective. Both mEGFP and GFP(S65T) fluorescently tagged proteins were imaged with a 488 nm laser at 30% power for single Z stacks and 20% power for movies.

All image analyses were done on either NIS elements or Fiji as previously described (Davidson et al., 2016; Gregory et al., 2025; Longo et al., 2022; Singh et al., 2025; Ye et al., 2025). Filament length was measured by drawing a line in Fiji along the length of the three longest filaments in individual cells. Fluorescence intensity was measured by drawing an ROI around the periphery of individual cells to measure the mean intensity. The intensities for all the measured cells were corrected for the background by subtracting the mean intensity from 50 *spn4Δ* cells. The brightness difference between mEGFP and GFP(S65T) was corrected by dividing the GFP(S65T) values by 0.91, which is the conversion rate calculated from each fluorophore's extinction coefficient and quantum yield (Cranfill et al., 2016; Patterson et al., 1997). The *p* value was calculated using unpaired two-tailed Student's t-test.

## Reagents

**Table:** 

**Strain name**	**Genotype**	**Figure/Movie**
JW81	*h- ade6-210 ura4-D18 leu1-32&nbsp;&nbsp;*	For gene targeting
JW295	*h* + *spn4Δ::kanMX6 leu1-32 ura4-D18*	Figure 1A, B, F
JW10492	*h- spn4-mEGFP:kanMX6 ade6-M210 leu1-32 ura4-D18*	Figure 1C, F
JW8589	*spn4-GFP(S65T):kanMX6 ade6 leu1-32 ura4-D18*	Figure 1C, F
JW10461	* h- kanMX6:81nmt1-mEGFP- spn4 ade6-210 ura4-D18 leu1-32 *	Figure 1A- F; Movie 1
JW311	* h- kanMX6:81nmt1-GFP(S65T)- spn4 ade6-M210 leu1-32 ura4-D18 *	Figure 1A- F; Movie 1
JW10474	* h- kanMX6:41nmt1-mEGFP- spn4 ade6-210 ura4-D18 leu1-32 *	Figure 1C, F
JW310	* h-kanMX6:41nmt1-GFP(S65T)- spn4 ade6-M210 leu1-32 ura4-D18 *	Figure 1C, F
**Plasmid name**	*&nbsp;*	&nbsp;
JQW75	pFA6a-kanMX6-P41nmt1-mEGFP	&nbsp;
JQW78	pFA6a-kanMX6-P81nmt1-mEGFP	&nbsp;
JQW85	pFA6a-mEGFP-kanMX6	&nbsp;

## Data Availability

Description: Movie 1. Time-lapse microscopy of the septin Spn4 in 81nmt1-mEGFP-spn4 and 81nmt1-GFP(S65T)-spn4 strains. The indicated strains were grown in YE5S for ~12 h, and then were washed 3x with EMM5S and grown exponentially in EMM5S for ~24 h at 25℃. These strains were imaged on an EMM5S + gelatin pad using a spinning disk confocal microscope every 30 sec for 1 h. Maximal intensity projection of 5 slices with 2 µm spacing are shown. 7 frames per second.. Resource Type: Audiovisual. DOI: https://doi.org/10.22002/e4bh4-a6g30
